# Continence care quality from the perspective of older adults in long-term care or in receipt of home care: a scoping review

**DOI:** 10.1136/bmjopen-2025-107685

**Published:** 2026-02-04

**Authors:** Anastasia Silverglow, Ian Milsom, Megan Kennedy, Helle Wijk, Adrian Wagg

**Affiliations:** 1 Institute of Health and Care Sciences, University of Gothenburg, Göteborg, Sweden; 2 Obstetrics and Gynecology, Sahlgrenska Academy, University of Gothenburg, Göteborg, Sweden; 3 Geoffrey and Robyn Sperber Health Sciences Library, University of Alberta, Edmonton, Alberta, Canada; 4 Health and Caring Sciences, Sahlgrenska Academy, University of Gothenburg, Göteborg, Sweden; 5 Obstetrics & Gynaecology, Sahlgrenska Academy, University of Gothenburg, Göteborg, Sweden; 6 Medicine, University of Alberta, Edmonton, Alberta, Canada

**Keywords:** Nursing Homes, Urinary incontinences, GERIATRIC MEDICINE

## Abstract

**Abstract:**

**Objectives:**

To assess the state of the research literature addressing what is known about the quality of continence care from the perspective of older adults in long-term care or in receipt of home care.

**Design:**

Scoping review of the literature according to the Joanna Briggs Institute method, reported according to Preferred Reporting Items for Systematic Review and Meta-Analysis Protocols guidelines. *Participant:* older adults (>65 years of age), either in receipt of home health or social care services or older adult residents of long-term care (nursing homes). *Concept:* older adult perspectives on quality of continence care (access, care to meet needs, continuity, goals, expectations, delivery, experiences, personalised care, partnerships in care, well-being and social support). *Context:* older adults in long-term care or in receipt of home care.

**Results:**

We identified 14 articles from the academic literature. Sources originated from the USA (7), Australia (4), Canada (2) and 1 from Italy. Long-term care residents were the focus of 12 of the articles. Older adults reported limited access and information regarding continence care and services and variable abilities of care staff to deliver care. Older adults wanted to be actively involved in decisions about their care, preserve their autonomy and independence and wanted care to enhance their well-being.

**Conclusions:**

Studies examining the perspectives of older adults regarding the quality of their continence care are few. Older adults value person-centredness, expert advice regarding their condition, allowing preservation of self-determination and independence where possible. Older people value meaningful relationships with empathetic care providers. There remains a need for education of care providers in continence care and for policies and practices to support continence in a dignity-preserving framework.

**Registration:**

Open Science Framework (https://osf.io/bprq9/).

**STRENGTHS AND LIMITATIONS OF THIS STUDY**

- Robust methodological approach to the gathering and synthesis of evidence.
- Broad scoping of traditional and grey literature.
- Theoretically framed approach to assessment of care quality.
- Mixed samples may have led to exclusion of relevant data.

## Background

 Urinary incontinence (UI) is a common condition in older adults; in women aged≥70 years, more than 40% are affected. Prevalence is even higher in older adults in receipt of home care and residing in long-term care.^1^,^3^

UI is a risk factor for social isolation and physical deconditioning, associated with depression, falls and early mortality and impaired quality of life. UI also increases the risk of institutionalisation for older adults in the community and has an immense economic burden for healthcare systems, which is largely generated by costs for the care of older people.^4 5^ For example, in Sweden, the annual cost of UI has been reported between 3.4 and 5.4 billion Swedish kronor.^6^ Likewise, in Canada, the cost of care for an older adult with UI residing in a long-term care facility is considerable, with estimates ranging between $C4000 and $C14 000 per year.^7^

Quality of care is an essential principle for healthcare systems and currently a top priority nationally and internationally.^8 9^ Patients’ perceptions are important indicators of care quality and play a crucial role in quality improvement work.^10 11^ From the patient’s perspective, quality of care can be defined as ‘the totality of features and characteristics of a healthcare product or services, that bear on its ability to satisfy the stated or implied needs of the consumers of these products or services’.^12^ Patient satisfaction can operationalise the quality-of-care concept and can be understood as the degree to which services meet patients’ needs in terms of the environment, structure, process and outcomes of their care.^13^

The quality of continence care is often assessed either in terms of adherence to generally accepted clinical guidelines^14^ or from the perspective of the care provider, either the organisation or front-line clinician.^15^,^17^ Whether this constitutes true ‘quality’ or measures what truly matters to end users is debateable.^18^ Care focused on care, rather than cure, and preservation of dignity in care during intimate care processes is important in the care of older adults. Surprisingly, the end user’s leading role as the recipient of care has seldom been assessed.^19^ There are few data which seek to define the quality of care, or desired outcomes of care, from the perspective of care recipients. Those that do exist are limited to specific diagnoses. For example, an exploration of desired outcomes in women receiving urogynaecological care^20^ which did not consider the quality of care. In a study of older adults living with dementia and their care partners, there was a lack of data from the older adults themselves.^21^ The perspectives of older adults, often those with multimorbidity or frailty, who may be either partially or fully dependent on others for help in their daily care, may well differ from those of more robust older adults. Implementation of ‘cure’ directed care may be completely inappropriate or misguided, given the circumstances in which the older adult lives. For example, in a qualitative study of continence care preferences of adults in a palliative care unit, patients opted for indwelling urethral catheters, which would be considered as inappropriate in current guidelines, but it ‘solved their problem’.^22^

There remains a need to explore what quality of continence care means from the perspective of medically complex and vulnerable older adults. This scoping review aims to assess the state of the research literature addressing this question from the perspective of older adults in long-term care and from those in receipt of home care to identify gaps in the literature and direct further research.

## Theoretical framing

This scoping review was framed around two key theories: that of quality of care from the perspective of the care recipient and the concept of person-centred care. Application of the core precepts of these theories guided the data extraction and synthesis in this scoping review (box 1). Data from included studies took these precepts into account in the data analysis and synthesis.

Box 1 **Theoretical framing**

**Quality of care**

Access and effectiveness absorb other attributes, such as continuity, appropriateness and timeliness, and reflect care recipients’ satisfaction and well-being.^81^ These were chosen as the core dimensions of quality of care within this scoping review.

**Access**

Access concerns healthcare access (geographical or physical). The structural aspects of availability, a common language and a broad range of care to meet individuals’ needs and continuity of care are also included.

**Effectiveness**

Effectiveness relates to clinical care in terms of the individuals’ health outcomes, health status and mortality. Interpersonal care, the patient–provider relationship and communication emphasise that quality of care requires considering individual circumstances and the complexity of each person’s life situation.^81^

**Person-centred care (PCC)**

PCC prioritises the person’s individual needs, preferences and values. PCC treats patients as equal partners in the care processes, involving them in their care, and takes into consideration their personal context, experiences and goals.^82 83^

A person-centred nursing framework^84^ was chosen to operationalise PCC. Care processes that enable shared decision-making for all involved in care, as well as establish an effective relationship between all care professionals, are supported.^85^

## Methods

This review followed the Joanna Briggs Institute (JBI) method for scoping reviews.^23^ The Preferred Reporting Items for Systematic Review and Meta-Analysis Protocols (PRISMA-ScR) checklist (online supplemental file 1) was used for reporting.^24^ The protocol has been published,^25^ but briefly, the review included research studies of any method, government reports, abstracts from scientific conferences, review articles that identify a method for the review (meta-synthesis, systematic, scoping and narrative reviews) that focused on older adults residing in long-term care (nursing homes) or in receipt of home healthcare services. Reports dealing with community-dwelling older adults not in receipt of home healthcare, or where the status of older adults included could not be discerned, were excluded.

The JBI concept model was employed: *Participant:* older adults (>65 years of age), either in receipt of home health or social care services or older adult residents of long-term care facilities (nursing homes). *Concept:* older adult perspectives on quality of continence care (including access, care to meet needs, continuity, goals, expectations, delivery, experiences, personalised care, partnerships in care, well-being, social support. *Context:* older adults receiving home care in any non-long-term care environment, older residents of long-term care (nursing homes).

A librarian scientist (MK) performed an initial search of MEDLINE (21 November 2023) to identify articles on the topic. The text words contained in the titles and abstracts of relevant articles, and the index terms used to describe the articles, were used to develop a full search strategy for Ovid MEDLINE (online supplemental table 1). The search strategy was then adapted for each included database. A second librarian scientist reviewed the strategy using the Peer Review of Electronic Search Strategies guideline.^26^ The search was updated prior to submission for publication (March 2025) to reflect newly published studies since the original run. Ovid MEDLINE, Ovid Embase, CINAHL, Scopus, Web of Science Core Collection, Cochrane Library (via Wiley) and ProQuest Dissertations & Theses Global served as the basis for the search. Studies published in any language were included. The reference list of all included sources of evidence was screened for additional studies. A grey literature search of unpublished literature, including publicly available information produced by all levels of government, academic institutions, professional societies, business and industry, in print and electronic formats, was also performed. A member of the research team (AS) reviewed the first two hundred results from Google Scholar for inclusion. General and targeted internet searches involved the use of Google, while targeted searches examined the websites of national and international organisations addressing this subject matter. Search returns were screened for relevance prior to inclusion in the later stages of source management as below.

**Figure 1:**
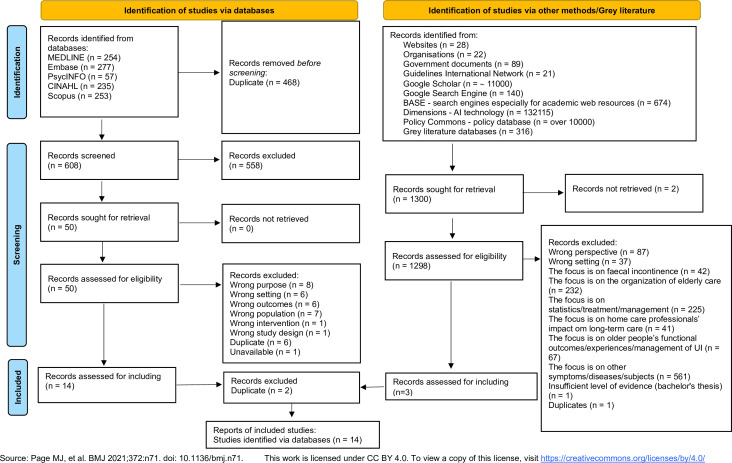
Preferred Reporting Items for Systematic Review and Meta-Analysis Protocols flow diagram. AI, artificial intelligence; BASE, Bielefeld Academic Search Engine; UI, urinary incontinence.

Search results were collated and uploaded into Covidence (Veritas Health Innovation, Melbourne); a web-based collaboration software platform that streamlines the production of systematic and other literature reviews. After automatic removal of duplicates, two reviewers independently screened titles and abstracts of a random sample of 5% of studies. Inter-reviewer agreement for inclusion or exclusion was assessed, revealing a kappa statistic of 0.94. The full text of included citations was assessed in detail against the inclusion criteria by two independent reviewers (AS and AW). Disagreements that arose were resolved through discussion and consensus between reviewers, and if needed, with a third party. The results of the search and the study inclusion process are presented in the PRISMA-ScR flow diagram (figure 1).

Data from included sources were extracted independently by two researchers (AS and AW). The information collected included the following details: date, country, authors, participants (number, sex, gender (if available), concept, context, study methods, elements of patient-defined quality, elements of person-centred care considered in the paper and key findings relevant to the review question. Data extracted and coded by the reviewers were then discussed with all authors to ensure agreement and consensus on relevant elements.

### Patient and public involvement

Patients or the public were not involved in the design, conduct, reporting or dissemination plans of the scoping review.

## Results

We identified 14 articles from the published literature, which originated from the USA (7), Australia (5), Canada (2) and 1 from Italy. The flow diagram is shown in figure 1 and the data extraction table, online supplemental table 2. Long-term care residents were the focus of 12 of the articles, the majority of which had a qualitative design and were published from 2000 to 2022; all were in English. The sample sizes ranged from 3 to 245, the latter being a clear outlier due to its record review method. Major findings are considered below with reference to our framework of access, effectiveness and person-centredness.

### Access to care

#### Information

Two studies^27 28^ highlighted the need for accurate information. Older people lacked information about resources, available support and costs for care and overall, expressed a desire for more information.^27^ They valued detailed information about referral, continence-specific services provided and help with accessing specific products. Older adults needed information that might allow them to take control of their problem. Lack of knowledge among care professionals about incontinence, inadequate resources and deficiency in specific skills of health team members may exacerbate older people’s reluctance to seek information from them. This may in turn result in poor care during transitions, particularly between the hospital and the community. Poor transitional care could result in absence or delayed referral on hospital discharge.^27^ Lack of information about schemes available to help defray the costs of continence products and equipment resulted in delayed access.^27^

When older people were dissatisfied with information received, they needed to take personal responsibility to find appropriate information. To achieve this, multiple sources of advice and information were used (library, via the Internet, books, pamphlets, special interest groups, by receiving personal explanation from care professionals and using advice from their family).^27 28^

#### Availability of care professionals, aids and adapted physical environment

11 studies reported that older people valued availability of care in terms of having access to practical resources,^2729^,^32^ an adapted physical environment^28 32^ and timely assistance of care professionals.^28^,^37^ Older adults appreciated having access to incontinence products such as pads, catheters, equipment, specialised clothing and protective items.^27 30 34^ The close proximity and availability of a clean bathroom^28 32^ and a call bell were needed to avoid major worry. Some residents who shared bathrooms wished to have a private bathroom.^28 32 34^

In six studies, older adults reported that they received fewer toileting assists than they preferred.^2833^,^37^ There were long delays between asking for and receiving help with toileting.^2829 33^,^37^ Timely delivery of containment products from care professionals,^31^ perceived lack of staff, particularly during the night^34^ and lack of timely care^28^ were reported. Anxiety and distress,^34^ the feeling of humiliation^33^ and a need to find alternative strategies for getting aids, for example, by only asking care professionals about absorbent pads and diapers at night, when other residents were sleeping,^31^ were mentioned in one study each.

#### Impact of cost

The cost of containment products was noted as a barrier to achieving preferred care in four studies.^27 28 32 38^ Older adults described incontinence products as expensive and a challenge to obtain in three.^27 28 38^ Costs varied in relation to the severity of incontinence, their financial situation and whether they additionally required surgery or medication. The cost of care was usually not an issue when there was public assistance. However, when private care was needed, costs could affect both care affordability and access, as well as result in gaps in care for people. Older people who experienced moderate to severe incontinence mostly bore the cost of incontinence aids and protective items, as well as those related to extra laundry and cleaning. These costs were hidden but were often perceived as more important than the cost of accessing care professionals.^27^ In one study, older people reported receiving help from their relatives in obtaining products or buying them at the store themselves.^28^ Financial rationing, payment status and personal finances could determine who could use which bathrooms and under what circumstances.^32^

### Effectiveness of care

#### Physical comfort

Five studies highlighted older people’s desire for physical comfort when using UI aids in terms of comfort and convenience,^28 29 31 38 39^ and two mentioned functional simplicity.^28 32^ Older people described that their life felt easier when they had easy-to-use products.^28^ Simplicity of use made older people with UI feel safe and confident,^28 32 38^ especially when they could manage an independent change of products. In one study, those who feared they would leak outside their continence garment felt unsafe and stayed in their room.^40^

In two studies, older people emphasised that a good UI aid with a good absorption capacity, shape and comfort level was very important for their physical comfort.^29 31^ However, some indicated that they had to use pads of a size that did not fit them.^28^

#### Feeling of well-being

Five studies described that quality of continence care was linked to a feeling of well-being, which was dependent on the care professionals’ attitudes, rituals and routines^30^,^3235 36^ and competence.^32^ Language used by the care professionals affected older people’s feeling of well-being because it was sometimes perceived as ageist^30^ or ‘potty mouth’ talk and ‘closed lip talk’. Care professionals’ attitudes towards physical safety, biological contamination and continence care routines had an impact on routine toileting, such as staff rounds, types of incontinence products, privacy for toileting^30^,^32^ and feeling of dignity.^31 32 35^ In two studies, continence care routines were perceived as being unadapted to older people’s needs or requests.^30 35^ In such situations, some older people expressed fear in asking care professionals for assistance. They experienced a loss of dignity, which was described in terms of having one’s body exposed, feeling objectified and having care professionals’ focus on completing a task instead of attending to residents’ care needs.^35^ In three studies, older adults emphasised that their feeling of well-being was linked to care professionals’ competence, e.g. knowledge about how to handle a catheter,^28 32 35^ and in one study, care continuity in terms of receiving care from care professionals who were aware of their needs was reported.^31^

In situations where older adults were dependent on technologies to facilitate care, e.g. a patient hoist or lift to get out of bed, well-being was linked to trust or uncertainty in relation to these technologies. For example, they could refuse to get up with the help of a patient lift because they were afraid of getting stuck.^37^

### Person-centredness of care

#### Listening to the narrative of the patient

Five studies noted that older people valued care professionals’ interest in and attention to their individual experiences of incontinence in terms of their awareness about the person as an individual.^34^ Creating relationships^33 40^ and discussion about individual needs and issues^27 28 34^ was considered important. Older people felt at ease with care professionals who were empathetic and compassionate.^27^ However, encounters with staff who lacked interest and/or empathy could result in them remaining silent despite suffering.^27 28^ Strong relationships between older people, their family members and care professionals were reported as an important prerequisite for effective management of incontinence.^33^ In one study, older people tried to co-create care management with care professionals by completing a brochure that included a description of his or her condition and preferences.^40^

#### Shared decision-making

10 studies demonstrated the importance of older people having the opportunity for shared decision-making in terms of self-determination,^28^,^3032 37^ discussion of how UI could be managed^27 33 37^ and having control in relation to their continence care.^30 31 34^ Maintaining self-determination in nursing homes was crucial, but older people sometimes felt that this was violated.^32^ For example, scheduled routines overrode personal preferences for care.^30^ Older adults tried to be as independent as possible,^29 37^ but they felt they were not supported or allowed to do so, for example, because of fall risks in the bathroom, which was a major source of dissatisfaction and could result in making choices that influenced their risk of an incontinence episode.^37^

Older adults indicated that they wanted to be ‘kept-in-the-loop’ about decisions regarding their continence care.^34^ They also emphasised the need to discuss the impact of UI on their situation and how it could be managed.^27^ However, they were generally uninvolved in making decisions about management strategies.^33^ Care professionals’ assessment of continence problems could differ markedly from the older person’s experience.^37^ Lack of decision-making and control was associated with an institutional culture that could devalue older people’s contributions to self-care.^30^ In one study, older co-operated with housekeeping, maintenance and nursing staff to obtain continence aids and accept help from relatives who could advocate for them.^34^

## Discussion

This scoping review focused on assessing what is known about the quality of continence care from the perspective of older adults in long-term care and from those in receipt of home care. It found only 14 studies published over 22 years.

### Access to care, information, availability of care professionals, incontinence aids and adapted physical environment, impact of cost

Older people with UI have insufficient information and access to resources or support to take control of their problems. Together with their reluctance to seek information from healthcare providers, these findings illustrate both older people’s vulnerability and their wish to take greater responsibility for their own continence care.^41^,^43^ The importance of maintaining older people’s independence is reinforced and is a well-recognised priority.^44^,^48^ However, independence requires that older adults have good access to evidence-based informed advice and support to maintain independence for as long as possible. Good communication and coordination of care services and information on relevant services, such as care pathways, are important.^49^

Although older people with UI appreciated the availability of care professionals when they needed help, access to continence products and a private bathroom, existing continence care didn’t meet their needs. Long delays between asking for and receiving help with toileting, lack of timely care and a lack of containment products can lead to anxiety, distress and humiliation. Older adults associated these issues with a lack of staff, as reflected in these reports.^1950^,^52^ There was a significant association between missed care and patient adverse events,^5053^,^55^ which calls for greater attention to continence care provided for older adults in these settings.

Older people in Australia and the USA reported that their continence care was affected by costs; expensive continence products, accessing care professionals and the ease of toilet access, which may suggest inequity in care access. Equal access to healthcare for the entire population is a strategic priority for the WHO.^56^ However, this principle can be challenged by the emergence of private healthcare actors, which may offer faster access to care services,^57^ and should be taken into account in policy discussion.

### Experience of care, comfort and well-being

UI can lead to social isolation^4^ and increase older people’s care dependency over time.^58^ Here, older adults with UI reported that aids that suited their needs, their body shape and that were simple to use promoted their independence. Greater attention should be paid here as the care recipient’s independence is associated with health satisfaction,^59^ social interactions^60^ and care dependency.^61 62^

Older people’s well-being may be threatened during continence care where care routines are not adapted to their needs. Care routines were often structured according to generally accepted clinical guidelines^14^ or from the perspective of the organisation or front-line clinical care provider, rather than the care recipient.^15^,^17^

Many older adults reported that the approach of care professionals affected their feeling of dignity in close-to-body care and in communication in relation to continence care. This may lead to them becoming fearful of asking care professionals for assistance. Older people associated this with care professionals’ insufficient competence and a lack of awareness of their needs. The importance of knowledge and skills which allow end user participation in care is well described.^4463^,^65^ Data appear to show that community nurses have insufficient knowledge in relation to effective care for older people with UI.^66^ Unfortunately, unregulated care professionals who provide the majority of hands-on care^67 68^ have had an education and training which has also been described as insufficient.^69^ This is a priority area on which to improve the quality of continence care in communities.

### Person-centredness of care

Older adults with UI value being listened to; they felt that this improved care professionals’ awareness of them as individuals, created relationships and allowed discussion of their needs. Relationships between older people and care professionals can be positively or negatively influenced by the attitudes and approaches of care professionals.^70^ Care professionals who lacked interest and/or empathy contributed to older people remaining silent despite suffering. This was reflected in the literature^44 71^ and highlights the importance of improvement, as ignoring or missing older peoples’ needs may lead to care-related safety risks.^72^

Shared decision-making is important for older people’s feeling of control, establishing trust, creating empowerment^44 73 74^ and is associated with self-determination.^44^ Maintaining self-determination in nursing homes may be violated by organisational routines. Existing clinical guidelines, except for those produced by the International Consultation on Incontinence^75^ on the assessment and management of incontinence, are seldom focused on the needs of frail older adults. Clinical guidelines often advocate that expectations and treatment should be adapted to needs, preferences and capacity of older adults, but do not discuss elements that older adults define as comprising quality.^76^,^78^ This means that there is a risk that older adults’ perspectives are not always taken seriously when planning care, a crucial aspect for person-centred care.^48 79^ A lack of shared decision-making and diminution of older people’s control is associated with a dominant organisational culture.^80^ Organisations that wish to support implementation of person-centred care should consider methods of giving legitimacy to the older adult’s perspectives in continence care.

### Strengths and limitations

The strengths of this study lie in the robust methodological approach to the gathering and synthesis of evidence. However, some studies included mixed samples of older adults, their relatives and their professional caregivers. The extraction of data that presented the older adults’ perspective sometimes needed interpretation by the research group. There is a risk that some relevant data from these studies were not included. Additionally, the majority of included studies comprised small samples, potentially limiting external validity. Only two studies examined the views of community-dwelling adults in receipt of home care, limiting conclusions drawn from that group.

### Knowledge gaps and future direction

The lack of home care situated studies highlights a significant gap in knowledge. Likewise, studies were exclusively performed in ‘western’ nations, revealing a gap in knowledge from older adults from other cultures, countries and in underserved groups of older adults, regardless of country.

No studies differentiated between UI subtypes or focused on faecal incontinence, where management may have differed and hypothetically, opinions on care quality may have differed. There also remains a need to assess the perspectives of modern nursing home populations, chiefly comprised of older adults with medical complexity, cognitive and physical frailty. These perspectives may differ from those of more cognitively able residents. There is a considerable opportunity for research to explore the views of differing groups of older adults but also further qualitative studies, mixed methods studies and co-created intervention studies which specifically incorporate the elements of quality highlighted here.

## Conclusion

Studies examining the perspectives of older adults regarding the quality of their continence care are few. Available evidence suggests that older adults value person-centredness, expert advice regarding their condition, allowing preservation of self-determination and independence where possible. Older adults value meaningful relationships with empathetic care providers who are willing to listen to their views and act on them when providing care. There remains a need for further research and for education of care providers in continence care and for specific policy frameworks designed to protect dignity during intimate care and models of care which support quality continence care in a dignity-preserving framework.

## Supplementary material

10.1136/bmjopen-2025-107685 online supplemental file 1

10.1136/bmjopen-2025-107685 online supplemental file 2

10.1136/bmjopen-2025-107685 online supplemental file 3

## Data Availability

Data sharing not applicable as no datasets generated and/or analysed for this study. Data are available upon reasonable request.
