# Supplementary material for: Continence care quality from the perspective of older adults in long-term care or in receipt of home care: a scoping review
Source: BMJ Open. 2026 Feb 4;16(2):e107685. doi: 10.1136/bmjopen-2025-107685 (PMC12878455; doi:10.1136/bmjopen-2025-107685)
Supplement: online supplemental file 2 [file bmjopen-16-2-s002.docx]

**Supplemental Table 1.** Database Search Strategies

All searches completed March 26th, 2025

Ovid MEDLINE(R) ALL <1946 to March 25, 2025>

1 exp Nursing Homes/ or exp Homes for the Aged/ or exp Rehabilitation Centers/ or exp Skilled Nursing Facilities/ or long-term care/ or ("nursing home*" or "extended care*" or "care home*").mp. or ((senior* or "continuing care" or disabled or "old age*" or geriatric* or "elder care*" or rehabilitat* or "long term care") adj2 (lodge* or facilit* or home* or residence* or centre* or center*)).mp. 125908

2 ((convalescen* or rest) adj2 home*).mp. 549

3 Assisted Living Facilities/ 1683

4 (("assisted living" or "assisted care" or "support* living") adj2 facilit*).mp. 2293

5 1 or 2 or 3 or 4 127580

6 exp Urinary Incontinence/ 37665

7 Fecal Incontinence/ 11302

8 ("urin* incontinen*" or "bladder incontinence" or "f?ecal* incontinen*" or "bowel incontinence" or "f?ecal soiling" or "continence care").mp. 60521

9 accidental bowel leak*.mp. 55

10 accidental* urine leak*.mp. 5

11 Urinary Bladder, Overactive/ 6409

12 (("overactive" or "over active") adj2 bladder*).mp. 10480

13 (urin* adj3 (incontinen* or involuntar* or leak* or uninten* or loss* or accident*)).mp. 56080

14 ((urgency or stress* or overflow or functional or mixed) adj3 incontinence).mp. 20882

15 OAB.ti,ab. 4402

16 ("bladder control*" adj4 (problem* or issue* or disorder* or dysfunction* or condition*)).mp. 75

17 urin* urge*.mp. 1672

18 ("urine drip*" or "urine dribbl*").mp. 50

19 bladder leak*.mp. 106

20 (incontinent or continent or continence).mp. 36997

21 ((F?eces or f?ecal or stool) adj3 (incontinen* or involuntar* or leak* or uninten* or loss* or accident*)).mp. 16746

22 or/6-21 100823

23 exp home care services/ or exp home nursing/ 52662

24 Independent Living/ 13736

25 (home care or residential care or domicil?iary care or "ag?ing in place" or communit* dwell* or independent* liv*).mp. 106395

26 23 or 24 or 25 118082

27 5 or 26 231644

28 exp qualitative research/ or grounded theory/ or exp Nursing Methodology Research/ or anthropology, cultural/ or exp focus groups/ or exp interview/ or exp interviews as topic/ or narration/ or (qualitative or ethnol$ or ethnog$ or ethnonurs$ or emic or etic or leininger$ or noblit or field note$ or field record$ or fieldnote$ or field stud$ or (participant$ adj3 observ$) or (nonparticipant$ adj3 observ$) or (non participant$ adj3 observ$) or hermeneutic$ or phenomenolog$ or lived experience$ or heidegger$ or husserl$ or merleau-pont$ or colaizzi$ or giorgi$ or ricoeur or spiegelberg$ or van kaam$ or "van manen" or (Grounded adj5 theor$) or (glaser and strauss) or ((content or theme* or thematic or narrative or discourse) adj2 analys*) or unstructured categor$ or structured categor$ or unstructured interview$ or semi-structured interview$ or semistructured interview$ or maximum variation or snowball or audiorecord$ or taperecord$ or videorecord$ or videotap$ or ((audio or tape or video$) adj5 record$) or ((audio$ or video$ or tape$) adj5 interview$) or meta-ethnog$ or metaethnog$ or meta-narrat$ or metanarrat$ or meta-interpret$ or metainterpret$ or (qualitative adj5 meta-analy$) or (qualitative adj5 metaanaly$) or ((interview* or audio* or video* or record*) and (views or perception* or belief* or attitude* or perspective*)) or purposive sampl$ or action research or focus group$ or photo voice or photovoice or mixed method* or ((audio* or video* or tape* or record*) and (interview* or transcri* or theme* or thematic))).mp. or ((metasynthes$ or meta-synthes$ or metasummar$ or meta-summar$ or metastud$ or meta-stud$ or hare or constant compar$ or theoretical sampl$ or triangulat$).ti,ab. or (experience*.ti. and (audio* or video* or tape* or record*).mp. and px.fs.)) 1116542

29 22 and 27 and 28 262

OVID Embase <1974 to 2025 March 25>

1 nursing home/ 66220

2 home for the aged/ 12028

3 rehabilitation center/ 20638

4 nursing home patient/ 9189

5 assisted living facility/ 3518

6 ("nursing home*" or "extended care*" or "care home*" or ((senior* or "continuing care" or disabled or "old age*" or geriatric* or "elder care*" or rehabilitat* or "long term care") adj2 (lodge* or facilit* or home* or residence* or centre* or center*))).mp. 136209

7 ((convalescen* or rest) adj2 home*).mp. 621

8 (("assisted living" or "assisted care" or "support* living") adj2 facilit*).mp. 4014

9 exp home care/ 95105

10 independent living/ 8813

11 ("home care" or "residential care" or "domicil?iary care" or "ag?ing in place" or "communit* dwell*" or "independent* liv*").mp. 153459

12 or/1-11 296836

13 exp incontinence/ or exp urine incontinence/ 125584

14 ("urin* incontinen*" or "bladder incontinence" or "f?ecal* incontinen*" or "bowel incontinence" or "f?ecal soiling" or "continence care").mp. 98577

15 accidental bowel leak*.mp. 135

16 accidental* urine leak*.mp. 5

17 overactive bladder/ 21251

18 (urin* adj3 (incontinen* or involuntar* or leak* or uninten* or loss* or accident*)).mp. 96008

19 (("overactive" or "over active") adj2 bladder*).mp. 23631

20 OAB.ti,ab. 9148

21 ((urgency or stress* or overflow or functional or mixed) adj3 incontinence).mp. 36653

22 ("bladder control*" adj4 (problem* or issue* or disorder* or dysfunction* or condition*)).mp. 122

23 urin* urge*.mp. 10475

24 ("urine drip*" or "urine dribbl*").mp. 80

25 bladder leak*.mp. 197

26 (incontinent or continent or continence).mp. 54699

27 ((F?eces or f?ecal or stool) adj3 (incontinen* or involuntar* or leak* or uninten* or loss* or accident*)).mp. 31401

28 or/13-27 193753

29 qualitative.mp. 521414

30 (ethnol$ or ethnog$ or ethnonurs$ or emic or etic).mp. 96465

31 (leininger$ or noblit or hare).ti,ab. 3398

32 (field note$ or field record$ or fieldnote$ or field stud$).mp. 32666

33 (participant$ adj3 observ$).mp. 27756

34 (nonparticipant$ adj3 observ$).mp. 213

35 (non participant$ adj3 observ$).mp. 1154

36 (hermeneutic$ or phenomenolog$ or lived experience$).mp. 63606

37 (heidegger$ or husserl$ or merleau-pont$).mp. 1613

38 (colaizzi$ or giorgi$).mp. 3032

39 (ricoeur or spiegelberg$).mp. 542

40 (van kaam$ or van manen).mp. 324

41 (Grounded adj5 theor$).mp. 24035

42 (constant compar$ or theoretical sampl$ or triangulat$).ti,ab. 22080

43 (glaser and strauss).mp. 158

44 (content analys$ or thematic analys$ or narrative analys$).mp. 138869

45 (unstructured categor$ or structured categor$).mp. 45

46 (unstructured interview$ or semi-structured interview$ or semistructured interview$).mp. 118800

47 (maximum variation or snowball).mp. 10206

48 (audiorecord$ or taperecord$ or videorecord$ or videotap$).mp. 146762

49 ((audio or tape or video$) adj5 record$).mp. 57589

50 ((audio$ or video$ or tape$) adj5 interview$).mp. 21644

51 (metasynthes$ or meta-synthes$ or metasummar$ or meta-summar$ or metastud$ or meta-stud$).ti,ab. 3100

52 (meta-ethnog$ or metaethnog$ or meta-narrat$ or metanarrat$ or meta-interpret$ or metainterpret$).mp. 1566

53 (qualitative adj5 meta-analy$).mp. 2167

54 (qualitative adj5 metaanaly$).mp. 22

55 purposive sampl$.mp. 21938

56 action research.mp. 7997

57 focus group$.mp. 91234

58 (photo voice or photovoice).mp. 1648

59 or/29-58 1006779

60 12 and 28 and 59 294

OVID APA PsycInfo <1806 to March 2025 Week 3>

1 exp nursing homes/ 10987

2 residential care institutions/ or assisted living/ or retirement communities/ 13304

3 rehabilitation centers/ 811

4 long term care/ 7191

5 ("nursing home*" or "extended care*" or "care home*" or ((senior* or "continuing care" or disabled or "old age*" or geriatric* or "elder care*" or rehabilitat* or "long term care") adj2 (lodge* or facilit* or home* or residence* or centre* or center*))).mp. 28929

6 ((convalescen* or rest) adj2 home*).mp. 138

7 assisted living/ 945

8 (("assisted living" or "assisted care" or "support* living") adj2 facilit*).mp. 980

9 home care/ 8237

10 aging in place/ or independent living programs/ 820

11 ("home care" or "residential care" or "domicil?iary care" or "ag?ing in place" or "communit* dwell*" or "independent* liv*").mp. 48172

12 or/1-11 76128

13 urinary incontinence/ 2231

14 fecal incontinence/ 697

15 ("urin* incontinen*" or "bladder incontinence" or "f?ecal* incontinen*" or "bowel incontinence" or "f?ecal soiling" or "continence care").mp. 3911

16 (("overactive" or "over active") adj2 bladder*).mp. 267

17 (urin* adj3 (incontinen* or involuntar* or leak* or uninten* or loss* or accident*)).mp. 3464

18 ((urgency or stress* or overflow or functional or mixed) adj3 incontinence).mp. 400

19 OAB.ti,ab. 164

20 ("bladder control*" adj4 (problem* or issue* or disorder* or dysfunction* or condition*)).mp. 29

21 urin* urge*.mp. 120

22 ("urine drip*" or "urine dribbl*").mp. 5

23 bladder leak*.mp. 0

24 (incontinent or continent or continence).mp. 2827

25 ((F?eces or f?ecal or stool) adj3 (incontinen* or involuntar* or leak* or uninten* or loss* or accident*)).mp. 1007

26 or/13-25 6723

27 qualitative study.md. 366833

28 exp qualitative research/ or grounded theory/ 43498

29 phenomenology/ or constructivism/ or hermeneutics/ 27890

30 ETHNOGRAPHY/ 12062

31 exp Content Analysis/ 11555

32 qualitative.mp. 261298

33 (ethno$ or emic or etic).mp. 50918

34 (leininger$ or noblit or hare).ti,ab. 1570

35 leininger m$.cu. 1774

36 noblit g$.cu. 1662

37 hare r$.cu. 14283

38 (field note$ or field record$ or fieldnote$ or field stud$).mp. 16303

39 (participant$ adj3 observ$).mp. 17319

40 (nonparticipant$ adj3 observ$).mp. 237

41 (non participant$ adj3 observ$).mp. 537

42 (hermeneutic$ or phenomenolog$ or lived experience$).mp. 83869

43 (heidegger$ or husserl$ or merleau-pont$).mp,cu. 20817

44 (colaizzi$ or giorgi$).mp,cu. 17657

45 (ricoeur or spiegelberg$).mp,cu. 7797

46 (van kaam$ or van manen).mp,cu. 5970

47 (Grounded adj5 theor$).mp. 28289

48 (constant compar$ or theoretical sampl$ or triangulat$).ti,ab. 15906

49 (glaser or strauss).mp. 3516

50 glaser b$.cu. 28420

51 strauss a$.cu. 54970

52 ((content or theme* or thematic or narrative or discourse) adj2 analys*).mp. [mp=title, abstract, heading word, table of contents, key concepts, original title, tests & measures, mesh word] 101667

53 (unstructured categor$ or structured categor$).mp. 32

54 (unstructured interview$ or semi-structured interview$ or semistructured interview$).mp. 74692

55 (maximum variation or snowball).mp. 4823

56 (audiorecord$ or taperecord$ or videorecord$ or videotap$).mp. 24842

57 (((audio or video*) adj5 (recorded or recording or tape* or taping)) or (tape adj3 record*)).mp. 26515

58 ((audio* or video* or tape* or taping or recording) and (interview* or transcri* or theme* or thematic)).mp. [mp=title, abstract, heading word, table of contents, key concepts, original title, tests & measures, mesh word] 34961

59 (metasynthes$ or meta-synthes$ or metasummar$ or meta-summar$ or metastud$ or meta-stud$).ti,ab. 1767

60 (meta-ethnog$ or metaethnog$ or meta-narrat$ or metanarrat$ or meta-interpret$ or metainterpret$).mp. 1196

61 (qualitative adj5 meta-analy$).mp. 450

62 (qualitative adj5 metaanaly$).mp. 3

63 purposive sampl$.mp. 8720

64 action research.mp. 13358

65 focus group$.mp. 54039

66 (photo voice or photovoice or mixed method*).mp. 45827

67 or/27-66 727911

68 12 and 26 and 67 60

CINAHL Plus with Full Text via EBSCOhost (1936 - Present)

S1 (MH "Nursing Homes+") 32,225

S2 (MH "Nursing Home Patients") 2

S3 (MH "Rehabilitation Centers") 9,214

S4 (MH "Long Term Care") 29,611

S5 ( "nursing home*" or "extended care*" or "care home*" ) OR ( ((senior* or "continuing care" or disabled or "old age*" or geriatric* or "elder care*" or rehabilitat* or "long term care") N2 (lodge* or facilit* or home* or residence* or centre* or center*)) ) 77,949

S6 ((convalescen* or rest) N2 home*) 253

S7 (MH "Assisted Living") 3,550

S8 (("assisted living" or "assisted care" or "support* living") N2 facilit*) 1,016

S9 (MH "Home Health Care+") OR (MH "Home Rehabilitation+") OR (MH "Home Respiratory Care+") 52,653

S10 (MH "Home Nursing") 3,965

S11 (MH "Community Living") 22,330

S12 ("home care" or "residential care" or "domicil#iary care" or "ag#ing in place" or "communit* dwell*" or "independent* liv*") 60,224

S13 S1 OR S2 OR S3 OR S4 OR S5 OR S6 OR S7 OR S8 OR S9 OR S10 OR S11 OR S12 199,248

S14 (MH "Incontinence+") 14,850

S15 ("urin* incontinen*" or "bladder incontinence" or "f#ecal* incontinen*" or "bowel incontinence" or "f#ecal soiling" or "continence care") 17,516

S16 "accidental bowel leak*" OR "accidental* urine leak*" 9

S17 (MH "Overactive Bladder") 2,172

S18 (MH "Urinary Incontinence+") 13,087

S19 (urin* N3 (incontinen* or involuntar* or leak* or uninten* or loss* or accident*)) 15,558

S20 ((overactive or "over active") N2 bladder*) 2,960

S21 TI OAB OR AB OAB 972

S22 ((urgency or stress* or overflow or functional or mixed) N3 incontinence) 5,185

S23 ("bladder control*" N4 (problem* or issue* or disorder* or dysfunction* or condition*)) 51

S24 "urin* urge*" 365

S25 ("urine drip*" or "urine dribbl*") 2

S26 "bladder leak*" 15

S27 (incontinent or continent or continence) 11,532

S28 ((F#eces or f#ecal or stool) N3 (incontinen* or involuntar* or leak* or uninten* or loss* or accident*)) 4,528

S29 S14 OR S15 OR S16 OR S17 OR S18 OR S19 OR S20 OR S21 OR S22 OR S23 OR S24 OR S25 OR S26 OR S27 OR S28 32,141

S30 (qualitative* OR ethnol* OR ethnog* OR ethnonurs* OR emic OR etic OR leininger OR noblit OR "field note*" OR "field record*" OR fieldnote* OR "field stud*" or "participant observ*" OR "participant observation*" OR hermaneutic* OR phenomenolog* OR "lived experience*" OR heidegger* OR husserl* OR "merleau-pont*" OR colaizzi OR giorgi OR ricoeur OR spiegelberg OR "van kaam" OR "van manen" OR "grounded theory" OR "constant compar*" OR "theoretical sampl*" OR (glaser AND strauss) OR "content analy*" OR "thematic analy*" OR narrative* OR "unstructured categor*" OR "structured categor*" OR "unstructured interview*" OR "semi-structured interview*" OR "maximum variation*" OR snowball OR audio* OR tape* OR video* OR metasynthes* OR "meta-synthes*" OR metasummar* OR "meta-summar*" OR metastud* OR "meta-stud*" OR "meta-ethnograph*" OR metaethnog* OR "meta-narrative*" OR metanarrat* OR " meta-interpretation*" OR metainterpret* OR "qualitative meta-analy*" OR "qualitative metaanaly*" OR "qualitative metanaly*" OR "purposive sampl*" OR "action research" OR "focus group*" or photovoice or "photo voice" or "mixed method*") 619,677

S31 S13 AND S29 AND S30 251

Scopus (1996 - Present)

( ( TITLE-ABS-KEY ( ( "nursing home*" OR "extended care*" OR "care home*" ) OR ( ( ( senior* OR "continuing care" OR disabled OR "old age*" OR geriatric* OR "elder care*" OR rehabilitat* OR "long term care" ) W/2 ( lodge* OR facilit* OR home* OR residence* OR centre* OR center* ) ) ) ) OR TITLE-ABS-KEY ( ( ( convalescen* OR rest ) W/2 home* ) ) OR TITLE-ABS-KEY ( ( ( "assisted living" OR "assisted care" OR "support* living" ) W/2 facilit* ) ) OR TITLE-ABS-KEY ( ( "home care" OR "residential care" OR "domicil?iary care" OR "ag?ing in place" OR "communit* dwell*" OR "independent* liv*" ) ) ) ) AND ( ( TITLE-ABS-KEY ( ( "urin* incontinen*" OR "bladder incontinence" OR "f?ecal* incontinen*" OR "bowel incontinence" OR "f?ecal soiling" OR "continence care" ) ) OR TITLE-ABS-KEY ( "accidental bowel leak*" OR "accidental* urine leak*" OR "bladder leak*" ) OR TITLE-ABS-KEY ( ( urin* W/3 ( incontinen* OR involuntar* OR leak* OR uninten* OR loss* OR accident* ) ) ) OR TITLE-ABS-KEY ( ( ( overactive OR "over active" ) W/2 bladder* ) OR oab ) OR TITLE-ABS-KEY ( ( ( urgency OR stress* OR overflow OR functional OR mixed ) W/3 incontinence ) ) OR TITLE-ABS-KEY ( ( "bladder control*" W/4 ( problem* OR issue* OR disorder* OR dysfunction* OR condition* ) ) ) OR TITLE-ABS-KEY ( "urin* urge*" OR "urine drip*" OR "urine dribbl*" ) OR TITLE-ABS-KEY ( ( incontinent OR continent OR continence ) ) OR TITLE-ABS-KEY ( ( ( f?eces OR f?ecal OR stool ) W/3 ( incontinen* OR involuntar* OR leak* OR uninten* OR loss* OR accident* ) ) ) ) ) AND ( TITLE-ABS-KEY ( ( qualitative* OR ethnol* OR ethnog* OR ethnonurs* OR emic OR etic OR leininger OR noblit OR "field note*" OR "field record*" OR fieldnote* OR "field stud*" OR "participant observ*" OR "participant observation*" OR hermaneutic* OR phenomenolog* OR "lived experience*" OR heidegger* OR husserl* OR "merleau-pont*" OR colaizzi OR giorgi OR ricoeur OR spiegelberg OR "van kaam" OR "van manen" OR "grounded theory" OR "constant compar*" OR "theoretical sampl*" OR ( glaser AND strauss ) OR "content analy*" OR "thematic analy*" OR narrative* OR "unstructured categor*" OR "structured categor*" OR "unstructured interview*" OR "semi-structured interview*" OR "maximum variation*" OR snowball OR audio* OR tape* OR video* OR metasynthes* OR "meta-synthes*" OR metasummar* OR "meta-summar*" OR metastud* OR "meta-stud*" OR "meta-ethnograph*" OR metaethnog* OR "meta-narrative*" OR metanarrat* OR " meta-interpretation*" OR metainterpret* OR "qualitative meta-analy*" OR "qualitative metaanaly*" OR "qualitative metanaly*" OR "purposive sampl*" OR "action research" OR "focus group*" OR photovoice OR "photo voice" OR "mixed method*" ) ) ) AND ( LIMIT-TO ( DOCTYPE , "ar" ) OR LIMIT-TO ( DOCTYPE , "re" ) )

Results: 282
