# Supplementary material for: Continence care quality from the perspective of older adults in long-term care or in receipt of home care: a scoping review
Source: BMJ Open. 2026 Feb 4;16(2):e107685. doi: 10.1136/bmjopen-2025-107685 (PMC12878455; doi:10.1136/bmjopen-2025-107685)
Supplement: online supplemental file 3 [file bmjopen-16-2-s003.docx]

| Author(s) | Year | Country | Method | Participants | Sample size | Concept | Context | Quality domain(s) | Person centred care elements | Key findings |
| --- | --- | --- | --- | --- | --- | --- | --- | --- | --- | --- |
| St John, W., James, H.,  McKenzie, S. [35] | 2002 | Australia | in-depth individual interviews, a  short structured questionnaire seeking demo-  graphic and descriptive information, and a focus  group | Independent adult clients known to be experiencing UI  Age (y)  30-39: 1  40-49: 2  50-64: 1  >64: 7 | 11 | clients’ perspectives of urinary continence service provision for com-  munity-dwelling people from a primary health care perspective | Home care | Access:  -professionals’ availability  -individuals’ needs and care continuity  -care to meet individuals’ needs  -cost  Efficiency:  -good or improved continence outcomes  -professionals’ knowledge | -professionals’ competence  -listening to the narrative of the patient  - effective relationship between all care professionals  - patients’ satisfaction and involvement with their care  - taking into consideration patients’ personal context, experiences, and goals | Appropriateness and Acceptability  Clients looked to “find the right person” and access accurate information, treatment, practical resources, good networks and services, and be able to discuss their individual issues. They valued practitioners who were knowledgeable, empathetic, interested in their experience of incontinence, compassionate, and with whom they felt at ease.  Accessibility and Intersectoral  Integration  Clients had lack of knowledge about incontinence, resources, and the specific skills of health team members. Many clients found out about specific continence services in a serendipitous way. Participants were often not aware of schemes available to help defray the costs of continence aids and equipment.  Equity and Affordability  Clients had lacked information about the availability of appropriate aids and support schemes, imposing both personal and financial costs.  INFORMATION  Participants overwhelmingly expressed a wish for more information. |
| O'Dell KK; Jacelon C; Morse AN [36] | 2008 | USA | semi-structured  interviews and a follow up visit to gather additional reflections | women with pelvic floor dysfunctions (PFD)  aged 65 to 96 | 25 | the views of  frail elderly women in residential care  related to QOL, values, and preferences for pelvic floor care | residential care facility | Access:  -physical access  - structural aspects of availability  -care to meet individuals’ needs  Effectiveness:  -individuals’ health outcomes | -consideration the patients’ personal context and experiences  - enable patients to derive satisfaction  - enhancing patients’ feeling of well-being | Close proximity and availability of a clean bathroom were very important to participants. Some of those who shared bathrooms reported this worked out well, but for most, it was a major worry  Disposable incontinence briefs were the most commonly preferred product. Women repeatedly lauded their comfort and convenience.  None of these residences provided briefs, and participants repeatedly described this style of product as expensive and a challenge to obtain.  Participants reported trying to be as independent as possible and minimize demands on staff.  While some women were able to toilet independently, others felt they could and were not allowed to because of fall risks in the bathroom. This restriction was a major source of dissatisfaction.  Some residents reported long delays between asking for and  receiving help, which occasionally resulted in urinary or fecal incontinence  Preferences for care varied among the participants (having catheter/ using pads/ pharmacologic treatments/surgery) |
| Johnson, Ouslander, Uman, Schnelle [37] | 2001 | USA | descriptive, comparative study. Interviews of older adults | nursing home residents | 79 | to describe preferences from  a variety of UI treatment options used in LTC | Nursing homes | Access:  -care to meet individuals’ needs  -respond in a timely  manner to requests for assistance  Effectiveness:  -individuals’ continence outcomes | Residents preferences should be taken into account where possible as proxies have markedly different views for care | Respondent groups commonly preferred options other than invasive strategies.  Respondents preferred medications to diapers.  Respondents wished to have access to practical resources and toileting assistance that meets their needs |
| MacDonald, Cathy D; Butler, Lorna [38] | 2007 | Canada | semi-structured interview | elderly women  with urinary  incontinence  age from 73 to 94 | 6 | experiences with UI while living in long-term care facilities | Nursing homes | Access:  -care to meet individuals’ needs  Effectiveness:  -inter-personal care  -communication | -involving residents in their care  - shared decision making  -prioritization the person's individual needs, preferences and values  -enhancing residents’ feeling of well-being  -care environment able to support good and safe care | Experiencing a  loss of control that was manifested in a number of ways such as loss of bodily control, loss of dignity, loss of independence.  Fear of embarrassing the staff providing them incontinence care  Encountering the Institutional Culture of UI in Long-Term Care  A culture existed, specific to the long-term care facility, in  which the women’s lives were influenced by rituals, routines, staff, and environment. Rituals and routines were mentioned by all the women when they spoke about the processes involved with routine toileting such as staff rounds, types of incontinence products, and allowable quotas, plus the lack of privacy for toileting.  The institutionalized culture affected how elderly women perceived themselves as well as their UI. Ageism seemed to be associated with the institutional culture and was evident in the lack of decision-making power and control these women had related to their incontinence care.  All of the participants acknowledged a lack of choice about types of products worn, toileting times, and changing of their incontinence products. Ageism was obvious in  the language used by the staff as they interacted about toileting, incontinence products, and UI.  The number of staff on a shift, gender, and attitudes of staff providing care directly affected the UI experience |
| Robinson, J.P. [39] | 2000 | USA | interviews and  participant observation with residents | Residents from 3 urban nursing homes | 10 English speaking older adult (>65y) residents | to develop a substantive theory describing and  explaining cognitive, psychosocial, and behavioural aspects of living with  urinary incontinence | Nursing homes | Access:  -respond in a timely  manner to requests for assistance  -care to meet individuals’ needs  Effectiveness:  -individuals’ continence outcomes | Personal preference – different strategies to hide incontinence and preserve sense of self  Negotiating individual solutions to management  Protection of self and autonomy | The residents invested in protecting physical and social integrity. They tried to preserve dignity and privacy despite UI.  The residents valued having information of how to prevent or manage leakage and  The residents used own strategies to manage actual or potential UI and suffered when these strategies were not supported by nursing staff. |
| Lyons, SAS  [40] | 2007 | USA | participant observation  and interviews | Residents with urinary  incontinence, families, and inter-disciplinary healthcare team members | unclear | to investigate organizational and  sociocultural aspects of continence care from the perspectives of nursing home  stakeholders | Nursing homes | Access:  -structural aspects of availability  Effectiveness:  -communication | -the person's individual needs, preferences, and values  - shared decision making | The organizational patterns in these facilities created situations in which residents did not have equal access to continence care.  Staff language patterns differed between facilities and set the stage for continence care interventions. Speech acts were classified as potty mouth talk, closed lip talk, and verbal resistance.  The residents endured situations in which their rights to privacy, dignity, and self-determination sometimes were violated during continence care. |
| Carsughi, Alessia; Santini, Sara; Lamura, Giovanni [41] | 2019 | Italy | Face-to-face semi-structured interviews | over-60-  year-old patients with UI | 8 older adults | study aiming at investigating the process of allocation, delivery and distribution of continence products  and at understanding the needs and expectations of community dwelling older patients with UI | Community dwelling older adults in receipt of containment products. (*Unable to discern if in receipt of other home care services)* | Access:  -structural aspects of availability  -care to meet individuals’ needs  -cost  Effectiveness:  -individuals’ continence outcomes | NA | Interviews noted the limited range of products and that these were unsuitable for many.  OA noted the need to buy their own products and that proffered services were seldom matched with need.  Many needed additional support service to mange continence, at cost.  Lack of education and support for OA with UI |
| Golenko X.; McLeod K.; Lowthian J. [42] | 2021 | Australia | semi-structured interviews | residents with incontinence  of mean age 87.4 years | 5 | staff and resident experience with assessment and management of incontinence  in a residential care home in south-east Queensland | residential care homes | Access:  -structural aspects of availability  -care to meet individuals’ needs  -respond in a timely  manner to requests for assistance  Effectiveness:  -communication  -inter-personal care | -involving residents in their care  -shared decision making  -enhancing the residents’ feeling of well-being  -partnership between patients and professionals | Residents were generally unaware that their continence and toileting needs were assessed; they did not have discussions with staff about their continence needs; and were generally not involved in making decisions about management strategies.  Response to calls for toileting assistance were not met in a  timely manner  Residents reported feelings of embarrassment, they felt reluctant to talk about incontinence, and felt humiliated when staff were unable to attend to their needs in a timely manner.  All participants  highlighted the importance of strong relationships between residents, family members and staff to  manage incontinence. |
| Ostaszkiewicz J, Cecil J, Kosowicz L, Garratt S, Wise E & Dow B [43] | 2022 | Australia | individual interviews | Patients (older people) with urinary  incontinence | 13 | aged care residents’ expectations and preferences for continence care in  Australian residential aged care homes | residential aged care homes | Access:  -receiving timely assistance  -care to meet individuals’ needs  -physical access  -individuals access the care they need  Effectiveness: NA | -partnership between patients and professionals  -listening to the narrative of the patient  -shared decision making  -receiving responsive assistance  -inter-personal care considering individual circumstances | Oh God, it’s come to this’  • How staff should deal with ‘it’  • Being kept in the loop  • Involving my family  Getting the help I need  • Receiving care from staff who care about me  • Being able to walk, but getting help when I need it  • Enough staff to care for me  • Being able to use incontinence products  • Having my own toilet and access to a call bell  • Being able to eat what I want |
| Ostaskiewicz,J. Ottman, G., Isaccs, R., Hutchinson, A. [44] | 2015 | Australia | Qualitative interviews | Residential aged care facility residents | Not stated | to explore key stakeholder  perspectives about ‘quality continence care’ in residential aged  care facilities | Residential aged care facilities | Access: respond in a timely and sensitive  manner to requests for assistance  Effectiveness:  -recognize diversity of resident’s care needs  -inter-personal care  -communication  -professionals’ competence  - adaptation of continence care routines to the residents’ needs | NA | Dignity preserving continence care was a major theme  Dignified continence care also meant being cared for by staff who convey compassion and empathy, offer choice.  QI interventions need to be contextually appropriate, in terms of accommodating the diversity of residents’ care needs, differences in staff knowledge, qualifications, roles and  responsibilities, and staffing levels and workloads |
| Schnelle, John F; Cadogan, Mary P; Grbic, Dragan; Bates-Jensen, Barbara M; Osterweil, Dan; Yoshii, June [45] | 2003 | USA | Medical  record reviews and short interviews | Residents with incontinence  70 to 94 | 245 | reliability and feasibility of a standardized protocol to assess and score urinary incontinence care in nursing homes | nursing homes | Access:  -structural aspects of availability  -care to meet individuals’ needs  Effectiveness: -- ---inter-personal care and communication | - involving the patients in their care | Residents received more or fewer toileting assists per  day than they preferred.  Residents perceived problems with frequency of toileting assistance and reported problems with the timeliness of assistance |
| Taunton, R.L., Swagerty, D.L., Lasseter, J.A., & Lee, R.H [46] | 2005 | USA | observation of resident care; abstraction of  resident records; and interviews with residents, | Residents ranged in age from 67 to 99 | 17 | the care provided to nursing home residents with urinary incontinence | nursing homes | Access:  -care to meet individuals’ needs  Effectiveness:  -considering individual circumstances and the complexity of each person’s life situation  -inter-personal care and communication | -involving residents in their care *(unclear whose perspective this was)* | Effective interpersonal skills elicit greater cooperation, especially important with residents who are cognitively impaired *(unclear whose perspective this was)*  Residents focused on “accidents.”  The episode of care is less stressful for the incontinent resident when sensitivity to  comfort is incorporated into the mechanical tasks, including perineal care. |
| O'Dell, Jacelon, Morse, McGee [47] | 2006 | USA | Qualitative semi structured interviews (conference abstract) | older women in assisted living  and long-term care facilities. | Twenty-five  women 65–96, mean 84 years | to increase  understanding of pelvic floor dysfunction (PFD) care  needs and preferences of older women in assisted living  and long-term care facilities. | Long term care and assisted living facilities | Access:  -cost of containment products noted as a barrier to achieving preferred care  Effectiveness:  -individuals’ continence outcomes | NA | Women expressed acceptance of PFD  on either the transition to residential care  or the choice of current activities. Concerns centered on  disturbance of sleep, embarrassment with leaking, lack of  access to bathrooms and preferred products, and discomfort with feeling “too wet.” |
| Jansen, Lynn; McWilliam, Carol L; Forbes, Dorothy; Forchuk, Cheryl [48] | 2013 | Canada | individual  interviews | home care recipients,  65 to 84 years | 3 | the social interaction processes related to how UI management knowledge might be translated within in-home care | home care | Access: NA  Effectiveness:  -inter-personal care and communication | -listening to the narrative of the patient  -shared decision making by creating a common understanding of the illness experience and planning care  -involving care recipients in their care | knowledge was created through social interaction processes: “We [caregiver and care recipient] refer to this [information shared by physicians and hospital staff prior to receiving home care] everyday to learn about my condition [UI and mobility issues] … We share this information with the home care people.”  home care recipients were supported through working relationships to be in control of and manage a chronic condition |

Supplementary table 2. Data extraction table
