# Supplementary material for: Continence care quality from the perspective of older adults in long-term care or in receipt of home care: a scoping review
Source: BMJ Open. 2026 Feb 4;16(2):e107685. doi: 10.1136/bmjopen-2025-107685 (PMC12878455; doi:10.1136/bmjopen-2025-107685)
Supplement: Supplementary file 4 [file bmjopen-2025-107685.reviewer_comments.pdf]

PEER REVIEW HISTORY

BMJ Open publishes all reviews undertaken for accepted manuscripts. Reviewers are asked to complete a checklist review form and are provided with free text boxes to elaborate on their assessment. These free text comments are reproduced below.

ARTICLE DETAILS

Title (Provisional)

Continence care quality from the perspective of older adults in long-term care or in receipt of home care: a scoping review

Authors

Silverglow, Anastasia; Milsom, Ian; Kennedy, Megan; Wijk, Helle; Wagg, Adrian

---

VERSION 1 - REVIEW

**Reviewer**	**1**
**Name**	**Blanker, Marco**
**Affiliation**	**University Medical Centre Groningen Thoraxcentre, department of General Practice and Elderly Care Medicine**
**Date**	**11-Sep-2025**
**COI**	**None**

In general, this is a relevant topic and a well performed scoping review. After rethinking and re-reading, I feel that my main concern lies with the length of the article and writing. The introduction is quite long and to be honest, I lost track. I'd suggest to make the introduction more concise. After formulating the aims, I believe the Methods section should start. The paragraphs 'theoretical framing' 'quality of care' and person-centred care' belong to the methods, but can also be placed in a box.

Further, the methods section is well detailed. No comments to that. The discussion is also too long to keep me interested as a reader.

Some other (minor) issues:

Authors report that sample size of the 14 studies ranged from 3 to 245. The 245 is a clear outlier, with the second largest study including 79 participants, and most studies including 25 participants or less.

Moreover, when discussing outcomes details on this, or on other aspects of the discussed articles are lacking. This makes it difficult to grasp the base of most statements.

On page 8, authors also mention the IUGA report, but it remains unclear to me how this report has incorporated patient views (the main goal of this review). Please clarify.

Page 9 - lines 31-35 are confusing. Although in this sentence, the number of reference totals 12 studies, it is incorrect to suggest that 12 studies reported on the three separate parts mentioned in this sentence.

Same holds for the "six studies" mentioned on page 10 - lines 41-43.

On page 11 - lines 12-15, the seven studies are not supported by seven references, and the same incorrect principle is present here.

The pattern is repetitive, I didn't list all sections where this occurred in the manuscript.

Given the small study samples of most studies, I doubt it - at all - the summary 'statistic' of the number of studies provides true information.

**Reviewer**	**2**
**Name**	**Sun, Zhijing**
**Affiliation**	**Peking Union Medical College Hospital**
**Date**	**24-Oct-2025**
**COI**	**None**

1. The review identifies a very limited number of studies, with a heavy emphasis on long-term care facilities and a notable lack of focus on home care settings (only 2 studies). The Limitations should explicitly acknowledge this imbalance as a significant constraint.

2. The review combines all forms of continence care without differentiating between subtypes of UI. In the Discussion, please acknowledge this as a limitation of the current evidence base and of the synthesis.

3. Strengthen the Discussion by linking the specific deficits identified to concrete recommendations for curriculum development and training focus areas.

4. Discussing the economic argument for investing in quality continence care

**Reviewer**	**3**
**Name**	**Mobasseri, Khorshid**
**Affiliation**	**Tabriz University of Medical Sciences**
**Date**	**25-Nov-2025**
**COI**	**None**

I thank the editors for the opportunity to review this important and timely scoping review. The topic is of significant clinical and policy relevance given global demographic trends and the high prevalence of incontinence in care-dependent populations. The manuscript is well-conceived and addresses a critical knowledge

gap. I recommend *Major Revision*, and my comments below are offered to strengthen the manuscript prior to publication.

Abstract

The results sentence: "Older adults reported limited access and information regarding continence care and services and variable abilities of care staff to deliver care, but they wanted care to preserve their autonomy and independence." The "but" is slightly awkward. Consider rephrasing, e.g., "Despite reporting limited access to information and variable care quality, older adults emphasized that their key priorities were the preservation of autonomy and independence."

In the search strategy, the term "older adult*" was used. For maximum sensitivity and alignment with standardized indexing, it is recommended to use the MeSH (Medical Subject Headings) term "Aged"

Add a keyword like "person-centered care" for better discoverability.

Introduction

The background is comprehensive and sets the stage well. No changes needed.

Methods

The results section effectively categorizes findings into the pre-defined framework of Access, Effectiveness, and Person-centredness. However, the connection between this structure and the stated use of a purely deductive thematic analysis is unclear. Please justify the choice of a deductive approach. I think a combination of deductive and inductive thematic analysis may have been used to generate the reported data.

language handling for non-English studies is good (using DeepL/Google Translate with native speaker checks), but no non-English studies were included—note if any were screened/excluded to confirm comprehensiveness.

In the methods section, explicitly state what kind of data or perspective was extracted from this grey literature source. Was it a report summarizing patient views, or was it used in a different way? If it does not directly report primary qualitative or quantitative data on patient perspectives gathered through research, its inclusion and how it contributed to the thematic analysis should be justified or reconsidered.

how the first 200 Google Scholor results were screened could be clarified to enhance reproducibility.

It is stated that the protocol was "published (48)" but this is redacted. For the final version, this citation must be included to ensure transparency.

The PRISMA-ScR flow diagram (Figure 1) is referenced but is not included in the provided manuscript text—ensure it is attached or embedded in the final submission, as it is essential for transparency.

Results

The manuscript states one article was identified from the grey literature (the IUGA patient website). However, the description of this source in the results (Ref 53) reads as an informational website for patients, not a study reporting primary data on patient perspectives. This needs clarification.

"Access to care, Information": The sentence "Older people lacked information about resources, available support and costs for care in some situations and overall, expressed a desire for more information (51)." The phrase "in some situations" is vague. Please specify what situations these were, if the source paper does so.

Page 14, The sentence "Many older adults reported that the approach of care professionals affected their feeling of dignity..." The word "Many" might be an overstatement given the limited number of studies. Consider "Several studies reported..." or "The included studies indicated that...".

In Access to Care," differentiate urinary vs. fecal incontinence where possible, as needs may differ.

The flow between paragraphs within the results sections could be slightly improved by using stronger concluding or linking sentences that summarize the main message of each sub-section before moving on.

Discussion and conclusion

Geographic representation is skewed (mostly USA/Australia), with no studies from low/middle-income countries. Discuss potential cultural biases in perceptions of dignity and person-centered care.

The review highlights a key finding: only 2 studies focused on home care recipients, limiting insights for this group. This imbalance is acknowledged in limitations but warrants more emphasis in the discussion (e.g., implications for "aging in place" policies). Consider recommending targeted future research on home care, perhaps stratified by frailty or dementia levels.

the discussion section could be better integrate findings with broader literature (e.g., how these align with WHO guidelines on integrated care for older people or other valid websites recommendations on frailty-specific continence care).

Economic aspects (e.g., costs as barriers) are underexplored—elaborate on equity implications, especially for underserved populations.

The discussion could be strengthened by adding a forward-looking paragraph on research implications. While the conclusion mentions the need for education and policy change, it should also issue a specific call for qualitative and mixed-methods studies in home care, inclusive research involving older adults with cognitive impairment, and co-designed intervention studies focused on aspects patients value, like communication and shared decision-making.

The conclusion would be strengthened by moving from general recommendations to more specific, actionable proposals. For instance, suggest specific educational interventions targeting unlicensed caregivers. Similarly, a recommendation for "policy change" could be refined to propose specific policy frameworks designed to protect dignity during intimate care.

VERSION 1 - AUTHOR RESPONSE

**Reviewer: 1**	
**Comment**	**Author's response**
1. In general, this is a relevant topic and a well performed scoping review. After rethinking and re-reading, I feel that my main concern lies with the length of the article and writing. The introduction is quite long and to be honest, I lost track. I'd suggest making the introduction more concise.	Thank you for the comment. We have edited the introduction accordingly
2. After formulating the aims, I believe the Methods section should start. The paragraphs 'theoretical framing' 'quality of care' and person-centred care' belong to the methods, but can also be placed in a box.	Thank you for the helpful comment. We have moved the theoretical framing section into a box, as suggested – "Box 1"
3. The discussion is also too long to keep me interested as a reader.	Thank you. We appreciate the comment and have endeavoured to ensure that the discussion is focused without losing the attention of the reader
4. Authors report that sample size of the 14 studies ranged from 3 to 245. The 245 is a clear outlier, with the second largest study including 79 participants, and most studies including 25 participants or less.<br>5. Moreover, when discussing outcomes details on this, or on other aspects of the discussed articles are lacking. This	Thank you. We have striven to ensure that where statements are made, that references to the data are clear. We have noted that most of the samples are small in the limitations section

makes it difficult to grasp the base of most statements.	
6. On page 8, authors also mention the IUGA report, but it remains unclear to me how this report has incorporated patient views (the main goal of this review). Please clarify.	Thank you for the comment. On reflection, we have deleted this report as it was for, rather than formed from, perspectives of older women
7. Page 9 - lines 31-35 are confusing. Although in this sentence, the number of reference totals 12 studies, it is incorrect to suggest that 12 studies reported on the three separate parts mentioned in this sentence.<br>8. Same holds for the "six studies" mentioned on page 10 - lines 41-43.<br>9. On page 11 - lines 12-15, the seven studies are not supported by seven references, and the same incorrect principle is present here.	Thank you – we have revised these sentences to make the meaning clear at each point.
10. Given the small study samples of most studies, I doubt it - at all - the summary 'statistic' of the number of studies provides true information.	We must apologise, the meaning of this comment is unclear. We have therefore been unable to respond appropriately. If this relates to the above comments, we have emended the content accordingly
**Reviewer 2**	
**Comment**	**Author's response**
1. The review identifies a very limited number of studies, with a heavy emphasis on long-term care facilities and a notable lack of focus on home care settings (only 2 studies). The Limitations should explicitly acknowledge this imbalance as a significant constraint.	Thank you for the comment. We believe that we have addressed this in the submitted paper but have reinforced this point
2. The review combines all forms of continence care without differentiating between subtypes of UI. In the Discussion, please acknowledge this as a limitation of the current evidence base and of the synthesis.	Thank you for the point. We have acknowledged that the evidence base seldom takes this into account, although care may not include active management which varies by UI subtype<br>"No studies differentiated Between UI subtypes, where management may have differed and hypothetically, opinions on care quality may have varied."
3. Strengthen the Discussion by linking the specific deficits identified to concrete recommendations for curriculum development and training focus areas.	Thank you. We have revised the discussion to address identified gaps accordingly
4. Discussing the economic argument for investing in quality continence care	Thank you for the interesting point. This is certainly an important area of focus although

	somewhat tangential to the aim of the paper. We have though acknowledged that this is an important area for investigation
**Reviewer 3**	
**Comment**	**Author's response**
1. The results sentence: "Older adults reported limited access and information regarding continence care and services and variable abilities of care staff to deliver care, but they wanted care to preserve their autonomy and independence." The "but" is slightly awkward. Consider rephrasing, e.g., "Despite reporting limited access to information and variable care quality, older adults emphasized that their key priorities were the preservation of autonomy and independence."	Thank you for the comment. We have revised the sentence accordingly for ease of reading. The sentence now reads "Older adults reported limited access and information regarding continence care and services and variable abilities of care staff to deliver care. Older adults wanted to play a part in their care, preserve their autonomy and independence and wanted care to enhance their well-being".
2. In the search strategy, the term "older adult*" was used. For maximum sensitivity and alignment with standardized indexing, it is recommended to use the MeSH (Medical Subject Headings) term "Aged"	Thank you for the comment. The search strategy was conducted by a librarian scientist with expertise. We are unable to redo the searches at this stage.
3. Add a keyword like "person-centered care" for better discoverability.	Thank you for the advice. We have done this
4. The results section effectively categorizes findings into the pre-defined framework of Access, Effectiveness, and Person-centredness. However, the connection between this structure and the stated use of a purely deductive thematic analysis is unclear. Please justify the choice of a deductive approach. I think a combination of deductive and inductive thematic analysis may have been used to generate the reported data.	Thank you for the comment. The reviewer is correct. We have amended the method accordingly
5. Methods<br>6. language handling for non-English studies is good (using DeepL/Google Translate with native speaker checks), but no non-English studies were included—note if any were screened/excluded to confirm comprehensiveness.	Thank you for the comment. We have clarified the fact that no non-English studies were located and that translation was not required

7. In the methods section, explicitly state what kind of data or perspective was extracted from this grey literature source. Was it a report summarizing patient views, or was it used in a different way? If it does not directly report primary qualitative or quantitative data on patient perspectives gathered through research, its inclusion and how it contributed to the thematic analysis should be justified or reconsidered.	Thank you for the helpful comment. We have removed the single grey literature source and the data extracted as, on reflection, the report did not gather data from women
8. how the first 200 Google Scholar results were screened could be clarified to enhance reproducibility.	This was simply a sequential process, and screening was sone by a single researcher. We have clarified this in the methods. The relevant content is now:<br>"A member of the research team (AS) reviewed the first two hundred results from Google Scholar for inclusion. General and targeted internet searches involved the use of Google, while targeted searches examined the websites of national and international organizations addressing this subject matter. Search returns were screened for relevance prior to inclusion in the later stages of source management as below."
9. It is stated that the protocol was "published (48)" but this is redacted. For the final version, this citation must be included to ensure transparency.	This was done to adhere to submission rules. This will be undone, and other redacted details reincorporated for the final version of the paper
10. The PRISMA-ScR flow diagram (Figure 1) is referenced but is not included in the provided manuscript text—ensure it is attached or embedded in the final submission, as it is essential for transparency.	Thank you . We apologise for the error, the PRISMA diagram was submitted as a separate figure and is now included with the submission package
11. The manuscript states one article was identified from the grey literature (the IUGA patient website). However, the description of this source in the results (Ref 53) reads as an informational website for patients, not a study reporting primary data on patient perspectives. This needs clarification.	Thank you for the comment, also noted by the other reviewer. AS noted, we have removed this grey literature source as the information was written for women rather than gathered from women.
12. "Access to care, Information": The sentence "Older people lacked information about resources, available support and costs for care in some situations and overall, expressed a desire for more information (51)." The	Thank you – on reflection we agree that this is vague and have rewritten the phrase

phrase "in some situations" is vague. Please specify what situations these were, if the source paper does so.	
13. Page 14, The sentence "Many older adults reported that the approach of care professionals affected their feeling of dignity..." The word "Many" might be an overstatement given the limited number of studies. Consider "Several studies reported..." or "The included studies indicated that...".	Thank you for the comment. We have revised the sentence accordingly
14. In Access to Care," differentiate urinary vs. fecal incontinence where possible, as needs may differ.	Thank you, we appreciate that there may well be differences between those living with either urine incontinence or faecal incontinence, or both. No paper made this distinction
15. The flow between paragraphs within the results sections could be slightly improved by using stronger concluding or linking sentences that summarize the main message of each sub-section before moving on.	Thank you for the comment. While rewriting the results section we hope we have improved the flow. We hope to summarize the messages from the results in the discussion section and have paid attention to this comment there.
16. Geographic representation is skewed (mostly USA/Australia), with no studies from low/middle-income countries. Discuss potential cultural biases in perceptions of dignity and person-centered care.	Thank you for the comment. We agree and have made reference to this as a potential limitation and a definite gap.
17. The review highlights a key finding: only 2 studies focused on home care recipients, limiting insights for this group. This imbalance is acknowledged in limitations but warrants more emphasis in the discussion (e.g., implications for "aging in place" policies). Consider recommending targeted future research on home care, perhaps stratified by frailty or dementia levels.	Thank you for this helpful suggestion . We have emended the content to reflect these points in the relevant sections
18. the discussion section could be better integrate findings with broader literature (e.g., how these align with WHO guidelines on integrated care for older people or other valid websites recommendations on frailty-specific continence care).	Thank you for the comments. We have noted that, although many clinical guidelines advocate that older adult's preferences for care, what might constitute quality in care from their perspective is often not included. Likewise, ICOPE focuses on intrinsic capacity, is aimed at primary care and consists of evidence informed intervention without discussion of quality.
19. Economic aspects (e.g., costs as barriers) are underexplored—elaborate on equity implications, especially for underserved populations.	Again, thank you for the comment. We have noted this as a knowledge gap

Commented [AW1]: @Anastasia - is this correct?

20. The discussion could be strengthened by adding a forward-looking paragraph on research implications. While the conclusion mentions the need for education and policy change, it should also issue a specific call for qualitative and mixed-methods studies in home care, inclusive research involving older adults with cognitive impairment, and co-designed intervention studies focused on aspects patients value, like communication and shared decision-making.	Thank you – we have rewritten the strengths and limitations section to incorporate identified gaps to point out potential directions for further research.<br>“There is considerable opportunity for research exploring the views of differing groups of older adults but also in further qualitative studies, mixed methods studies and co-created intervention studies which specifically incorporate elements of quality highlighted here.”
21. The conclusion would be strengthened by moving from general recommendations to more specific, actionable proposals. For instance, suggest specific educational interventions targeting unlicensed caregivers. Similarly, a recommendation for "policy change" could be refined to propose specific policy frameworks designed to protect dignity during intimate care.	Thank you for the helpful comment. We have rewritten the conclusion to make these points<br>“There remains a need for further research and for education of care providers in continence care and for specific policy frameworks designed to protect dignity during intimate care and models of care which support quality continence care in a dignity preserving framework”.

VERSION 2 - REVIEW

Reviewer 1

Name Blanker, Marco

Affiliation University Medical Centre Groningen Thoraxcentre, department of General Practice and Elderly Care Medicine

Date 08-Jan-2026

COI

Authors have substantially improved their manuscript based on the comments of three reviewers. My main concern remains with the length of this paper. It is (even with interest in the topic!) difficult to stay tuned. Although the introduction has been shortened, I feel that it could even be limited to 50% of the current text without losing relevant information.

VERSION 2 - AUTHOR RESPONSE

Reviewer: 1
Dr. Marco Blanker, University Medical Centre Groningen Thoraxcentre

Comments to the Author:
Authors have substantially improved their manuscript based on the comments of three reviewers. My main concern remains with the length of this paper. It is (even with interest in the topic!) difficult to stay tuned. Although the introduction has been shortened, I feel that it could even be limited to 50% of the current text without losing relevant information.

We thank the editors for the review of our revised manuscript. We have as suggested by Marco, shortened the paper as much as is possible without losing relevant information.

We have pleasure in returning a second revision where the text has been reduced accordingly.
